# Nepali translation, cross-cultural adaptation and measurement properties of the Shoulder Pain and Disability Index (SPADI)

**DOI:** 10.1186/s13018-019-1285-8

**Published:** 2019-08-30

**Authors:** Sudarshan KC, Saurab Sharma, Karen Ginn, Tawfiq Almadi, Darren Reed

**Affiliations:** 1 Discipline of Anatomy and Histology, Faculty of Medicine and Health, University of Sydney, Sydney, Australia; 2 Department of Physiotherapy, Kathmandu University School of Medical Sciences, Dhulikhel, Kavre Nepal; 3 Department of Surgical Sciences, Dunedin School of Medicine, University of Otago, Dunedin, Otago New Zealand

**Keywords:** SPADI, Shoulder pain, Disability, Pain, Translation, Psychometrics, Clinimetrics, Outcome assessment

## Abstract

**Background:**

The Shoulder Pain and Disability Index (SPADI) is a 13-item shoulder-specific patient-reported outcome measure (PROM). The English version is easy to use and has demonstrated excellent measurement properties for both clinical and research settings. The availability of the SPADI in Nepali would facilitate shoulder research and enhance management of patients with shoulder pain in Nepal. Therefore, the purpose of this study was to translate and cross-culturally adapt the SPADI into Nepali (SPADI-NP) and evaluate its measurement properties.

**Methods:**

The translation and adaptation process followed international guidelines. Participants completed SPADI-NP on two assessments (*N* = 150 at initial and 119 at follow-up assessment). A Nepali version of the Global Rating of Change score was completed at follow-up. Assessment of measurement properties included analysis of internal consistency (Cronbach’s α), minimal detectable change (MDC) with standard error of measurement (SEM), test-retest reliability (intraclass correlation coefficient; ICC), validity (factor structure, construct using Pearson’s correlation with the Disability of Arm and Hand [DASH]) and responsiveness (area under the curve; AUC) with minimal important change (MIC).

**Results:**

Minor changes were integrated in the adaptation process to improve cultural relevance such as dress items. Items were largely loaded under two factors (pain and disability), internal consistencies were good for the pain construct (*α* = 0.82) and disability (*α* = 0.88) and test-retest reliability was excellent (pain = 0.89, disability = 0.96). MDC was 5.7 (out of 100) with SEM = 2.1. Strong associations with the DASH (*r* = 0.63 pain, *r* = 0.81 disability) demonstrated its construct validity. The AUC was 0.68 and MIC was 12.3 (out of 100).

**Conclusion:**

The Nepali version of the SPADI demonstrated excellent reliability and validity. It can be used for the assessment of shoulder pain and disability in patients with shoulder pain in Nepal in both clinical practice and research.

## Introduction

Health-related patient-reported outcome measures (PROMs) are an important component of clinical assessments, providing the patients’ perspective of their health status and functional capacity. PROMs can consequently direct treatment and provide valuable feedback of progress of clinical conditions. They are also increasingly used as primary outcome measures in research. For valid use in different language groups, PROMs must not only be translated but also cross-culturally adapted, and measurement properties (validity, reliability and responsiveness) confirmed [1].

The Shoulder Pain and Disability Index (SPADI) is one of the most commonly used shoulder-specific heath status questionnaires [2] and has been described as the most responsive shoulder pain and disability tool for shoulder conditions [3]. It has also been ranked as one of the most relevant questionnaires by shoulder patients being easy to complete and the least time consuming [4]. The SPADI consists of 13 items and is divided into 2 constructs: pain (5 items) and functional disability (8 items). The SPADI was originally developed using a visual analogue scale (VAS) [5] but later modified to use a numerical rating scale to increase its versatility for interview or self-reporting administration [6]. The original English version has been shown to have excellent measurement properties in various shoulder conditions [2]. It has subsequently been translated and cross-culturally adapted into multiple languages (e.g. Norwegian, Turkish, Danish) with acceptable measurement properties [7]. However, it has not been translated into Nepali and there is currently no shoulder-specific PROM available in the Nepali language. Translation of an easy to use, versatile shoulder outcome measure such as the SPADI into Nepali would benefit Nepali therapists and medical practitioners in the clinical assessment of shoulder conditions and facilitate ongoing shoulder research in Nepal.

Therefore, the aims of this study were to (1) translate and cross-culturally adapt the SPADI into Nepali, (2) determine the measurement properties of the translated version and (3) compare the efficacy of administering the questionnaire by interview or self-completion.

## Methods and materials

The study was completed in two phases: translation and adaptation of the English version of the SPADI as recommended by the American Association of Orthopaedic Surgeons (AAOS) Outcomes Committee [8] and measurement property testing of the Nepali version of the SPADI as per the COnsensus-based Standards for the selection of health Measurement INstruments (COSMIN) checklist [9]. The permission to translate the SPADI into Nepali was granted by the original developer. The Institutional Review Committee of Kathmandu University School of Medical Sciences, Dhulikhel, Nepal, approved the study protocol.

### Participants

A sample of 150 participants was set as a target to meet the recommended number of participants to test the measurement properties of a PROM [10]. The inclusion criteria for participation were current shoulder pain, aged over 18 years and able to speak Nepali fluently. Shoulder pain was referred to as pain over the shoulder and/or antero-lateral aspect of the upper arm, which was aggravated by shoulder movements. Participants also had to test positive to one of the following clinical tests: Hawkin’s impingement test, Neer’s impingement test or resisted isometric manual muscle tests (external rotation and abduction). In order to capture a representative sample from both rural and urban areas of Nepal, the physiotherapy outpatient department of three hospitals (a not-for-profit community-based hospital, a general urban hospital and a large orthopaedic hospital) were used to recruit participants. The participants were provided with verbal information about the study and a written participant information statement in Nepali, which was read to illiterate participants. Literate participants signed their informed consent, and illiterate participants’ consent was gained verbally and signed by a witness.

### Instruments

#### Shoulder Pain and Disability Index (SPADI)

The 13-item tool (5 pain, 8 disability) SPADI is scored on an ordinal scale from 0 (no pain/no difficulty) to 10 (worst pain imaginable) with higher scores indicating greater pain and/or disability. To obtain a score out of 100, the total scores of all items are summed and divided by the highest possible score (130) and then multiplied by 100. If more than two items are left blank, the scoring is considered invalid [5].

#### Nepali version of the Global Rating of Change (GROC-NP)

The GROC assesses the self-perceived change of the participant’s health condition. It is commonly used in research and is the most common tool used to dichotomise shoulder pain sufferers into stable and changed groups [11]. The Nepali version of the GROC is based on a 7-point scale, which has been translated into Nepali [12] and has been used as an external anchor to dichotomise improved and stable groups previously [12–14]. The middle score of ‘4’ in the scale indicates no change in symptoms (stable), scores higher than 4 indicate progressive increments of improvement (slight, moderate and large) and a score lower than 4 indicates worsening symptoms (slight, moderate and large). A change of 1 point is considered important change [12, 15].

#### The Nepali version of Disability of Arm, Shoulder and Hand (DASH-NP)

The DASH is a 30-item scale measuring patient-reported pain and functional limitations. The Nepali version of the DASH has been cross-culturally translated and adapted and shown to be valid, reliable and responsive [16]. Each item is scored on a 5-point Likert scale giving a total score calculated out of 100, with higher scores indicating greater pain and disability. If more than three items are left blank, it is considered invalid.

### Phase I—Translation procedures and cross-cultural adaptation

Translation and cross-cultural adaptation were completed in five steps.

- Three forward translations of the original SPADI were independently created by a health professional, a non-medical person and a professional translator registered with and accredited by the National Accreditation Authority for Translators and Interpreters Ltd., Australia. All were bilingual, born in Nepal and native Nepali speakers. A written report was submitted by each translator outlining difficulties in the translation process.
- The three forward translations were then synthesised into a single version by the study co-ordinator (SKC) and two university academics (DR and SS), all bilingual and native English or Nepali speakers. Discrepancies in the translations were discussed and a consensus reached for back-translation.
- Two bilingual, native English-speaking translators, naive to the study aims, independently back-translated the synthesised version of the SPADI and submitted a written report suggesting any difficult or unclear phrases.
- An expert committee, comprising all translators, the principal investigator (SKC) and two university academics (DR, SS) reviewed the translated versions and discussed discrepancies/difficulties highlighted during the translation process. Consensus was reached within the expert committee, and a pre-final Nepali version of the SPADI (SPADI-NP) was produced.

The first five study participants completed the pre-final version of SPADI-NP and were interviewed using prepared questions to assess the layout, the wording of phrases and the ease of understanding of the questionnaire (comprehensibility). Majority consensus was used to accept phrases where more than one viable option was proposed. The data from these five participants were not retained for further analysis. This process led to the final version of the SPADI-NP.

#### Procedures

At the initial assessment, participants were provided with an information sheet, consent form, SPADI-NP and a validated Nepali version of the DASH-NP. The SPADI-NP was administered on a second occasion 2–3 weeks post-initial assessment along with the GROC-NP. Literate participants completed the SPADI-NP as a self-reported measure. Illiterate participants were interviewed by the physiotherapist who read the questionnaire to participants asking them to verbally select the most appropriate score without any prompting. If participants were unable to return to the hospital for follow-up due to remote living, a phone interview was used to collect follow-up data. All interviews and testing were performed by physiotherapists trained in the administration of the SPADI and data management procedures.

### Phase II—measurement property testing

Data was entered into an excel spreadsheet and later transferred to SPSS version 24 for statistical analyses.

#### Factor analysis

Suitability of data for factor analysis was assessed using correlations between the scale items with a threshold set at 0.40 [17], a Kaiser-Meyer-Okin (KMO) sample adequacy index requirement > 0.90 and a significant Bartlett’s sphericity test. A principal component analysis with varimax rotation was performed to examine the component structure of the 13-item SPADI-NP. Factors were retained if they had eigenvalues > 1. All eigenvalues were plotted on a Cattell’s Scree plot [18]. Items with loadings above 0.40 were assumed to load on a given factor.

#### Internal consistency

Internal consistency was analysed within the two constructs of pain and disability and as a total using Cronbach’s alpha (*α*) [19]. Scores between 0.50 and 0.69 were considered poor, 0.70 and 0.79 acceptable, 0.80 and 0.89 good and > 0.90 excellent [20].

#### Test-retest reliability

Test-retest reliability was determined using intraclass correlation coefficient (ICC_2,1_) between the initial and follow-up SPADI-NP scores of the participants in the stable group. Scores of < 0.40 were considered poor correlation, 0.40–0.59 fair, 0.60–0.74 good and > 0.75 excellent [19].

#### Measurement error

*Minimal detectable change* (MDC) was calculated using the formula MDC = *z* × √2 × SEM, where *z* = 1.96 (*z* score for estimating a 95% confidence interval), √2 indicates the two SPADI-NP measurements and SEM is the standard error of measurement calculated using the formula; SEM = SD (1 − ICC)^1/2^, where SD is the standard deviation for the mean change of SPADI-NP score from baseline to follow-up measurement and ICC is the intraclass correlation coefficient of the stable group.

#### Construct validity

Correlation between the pain items and disability items of the SPADI-NP and DASH-NP and the total SPADI-NP and the GROC-NP scores were calculated using Pearson’s correlation. We hypothesised that there would be a moderate to high positive correlation between the SPADI-NP and DASH-NP items and a moderate negative correlation with the GROC-NP. Coefficients < 0.35 represented low, 0.36–0.67 moderate, 0.68–0.89 high and ≥ 0.90 very high correlations [21].

Independent *t* tests were performed to compare the mean scores of participants completing the SPADI by self-marking with participants who were interviewed. Those completing the questionnaire by interview were further sub-grouped at the follow-up assessment, and an independent *t* test was used to compare those interviewed by phone with those interviewed at the hospital. Our a priori hypothesis was that there would be no difference in mean score between self-marking and any of the interview categories. The SPADI-NP would be considered to have acceptable construct validity if a minimum of four out of these five (i.e. 80%) a priori hypotheses were met [22].

#### Responsiveness

Receiver operating characteristic (ROC) curves were plotted. The area under the curve (AUC) was used to measure the SPADI’s ability to distinguish between these two groups and provide an indication of responsiveness [11]. An AUC > 0.70 was considered acceptable [22]. The minimal important change (MIC) was determined from the ROC curves.

## Results

### Participants

A description of the participant demographics is shown in Table 1. One hundred and fifty-six participants (75 M, 81 F; 47.7 ± 13.5 years) were recruited from the three physiotherapy outpatient departments. After exclusion of invalid questionnaires with > 2 items left blank (six at initial assessment and two at follow-up, total 3%), 150 completed SPADI-NP questionnaires were available from the initial assessment (66 by interview, 84 by self-report) and at follow-up assessment 119 (79%; 84 by interview over the phone, 25 by interview at a hospital, 10 by self-reporting). Thirty-one participants were categorised as “stable” with a GROC-NP score of 4 (15 of these completed both assessments by interview, 15 by self-report then interview and 1 by self-report at both assessments). Eighty-eight participants were categorised as “improved” with a GROC-NP score of 5, 6 or 7. No participant reported a GROC-NP score < 4 or worsening.

**Table 1:** Demographic description of participants

Items	*n* (%)	Mean ± SD
Gender		
Male	75 (48%)	
Females	81 (52%)	
Age		47.7 ± 13.5 years
Education		
No education	70 (45)	
Primary	58 (37)	
Secondary	14 (9)	
Bachelor and above	14 (9)	
Occupation		
Business	27 (17)	
Office work	17 (11)	
Agriculture	11 (7)	
Students	6 (4)	
Others (inc. house work)	95 (61)	
Ethnicity		
Brahmin	43 (27)	
Newar	26 (17)	
Chettri	20 (13)	
Others	67 (43)	
Religion		
Hindu	93 (60)	
Buddhist	34 (22)	
Others	29 (18)	

### Translation and cross-cultural adaptation

The following three modifications were made to the wording of the SPADI-NP during the translation and cross-cultural adaptation process. ‘10 pounds’ was changed to ‘5 kg’ as the metric system is used in Nepal; ‘pants’ was changed to ‘pants or *suruwal*’ as *suruwal* are the traditional pants both worn by both males and females; and ‘jumper’ changed to ‘sweater’ as ‘sweater’ is commonly worn by both sexes. The pilot testing revealed no difficulty with the understanding (comprehensibility) of all 13 items of the SPADI-NP. Two rural participants commented that they had trouble relating the numerical score to their pain and disability levels as this is an uncommon concept in remote Nepal. The final version of SPADI is located in the Appendix.

### Measurement properties testing

**Fig. 1:**
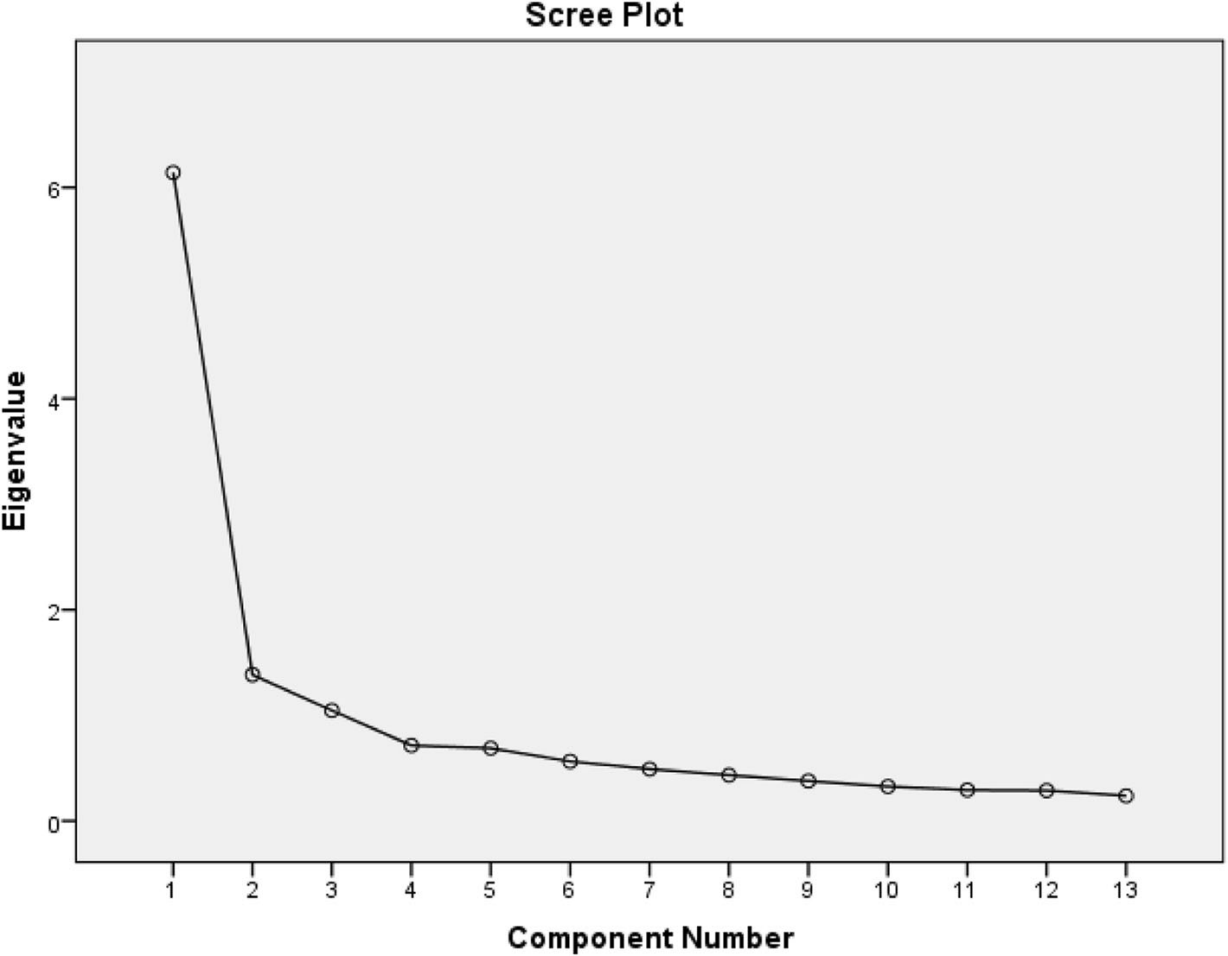
The Scree plot of items of SPADI-NP (components with high eigenvalues, > 1.0)

*Factor analysis*—data was determined to be suitable for factor analysis with many item correlation coefficients above the threshold of 0.40, a KMO sample adequacy index > 0.90 and a significant Bartlett’s sphericity test. Principal component factor analysis with varimax rotation produced loading of items in the two factors of pain and disability (Table 2) with a few items highly loading in both factors (item 3—reaching for something on a high shelf?, item 4—touching the back of your neck?, item 5—pushing with the involved arm? Item 12—carrying a heavy object of 5 kg?). The Scree plot (Fig. 1) shows a break after the second component, indicating two components being retained for the varimax rotation.

**Table 2:**
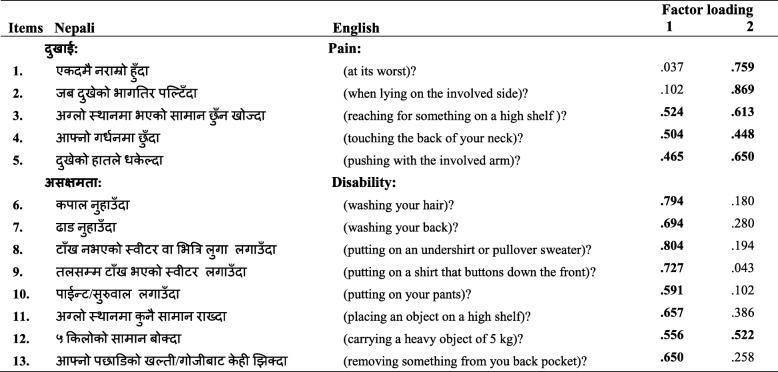
Principal component factor analysis with varimax rotation for individual items

Extraction Method: Principal Component Analysis. Rotation Method: Varimax with Kaiser Normalisation. Rotation converged in 3 iterations

*Internal consistency* (Cronbach α) and *test-retest reliability* (ICC) results are shown in Table 3 and revealed acceptable values for both internal consistency and test-retest reliability.

**Table 3:** Reliability and validity measures of the SPADI-NP

Measurement properties	SPADI—pain	SPADI—disability	Total SPADI-NP
Internal consistency (Cronbach’s α)	0.82	0.88	0.90
Test-retest reliability ICC (95%CI)	0.89 (0.80–0.95)	0.96 (0.92–0.98)	0.95 (0.90–0.97)
MDC			5.7 (out of 100)
SEM			2.1 (out of 100)
Construct validity			
DASH-NP—pain	*r* = 0.63*		
DASH-NP—disability		*r* = 0.81*	
GROC-NP			*r* = −0.41*

*CI* confidence interval, *DASH-NP* Nepali Disability of Arm, Shoulder and Hand score, *GROC-NP* Nepali Global Rating of Change, *ICC* intraclass correlation coefficient, *MDC* minimal detectable change, *SEM* standard error of measurement, *SPADI-NP* Nepali Shoulder Pain and Disability Index

*Significance *p* < 0.001

*Construct validity* analysis results are also included in Table 3 and confirm the five a priori hypotheses of:

- Moderate positive correlation between SPADI-NP and DASH-NP pain items (*r* = 0.63)
- High positive correlation between the SPADI-NP and DASH-NP disability items (*r* = 0.81)
- Moderate negative correlation between the mean change of the SPADI-NP scores and GROC-NP scores (*r* = − 0.41)
- Similar mean scores for participants completing SPADI-NP by self-report or interview (45.2 ± 23.8 and 46.1 ± 24.3, *p* = 0.82)
- Similar mean scores for participants completing SPADI-NP by face-to-face interviews at a hospital or over the telephone (45.6 ± 23.8 and 48.0 ± 24.4, *p* = 0.67).

### Responsiveness

**Fig. 2:**
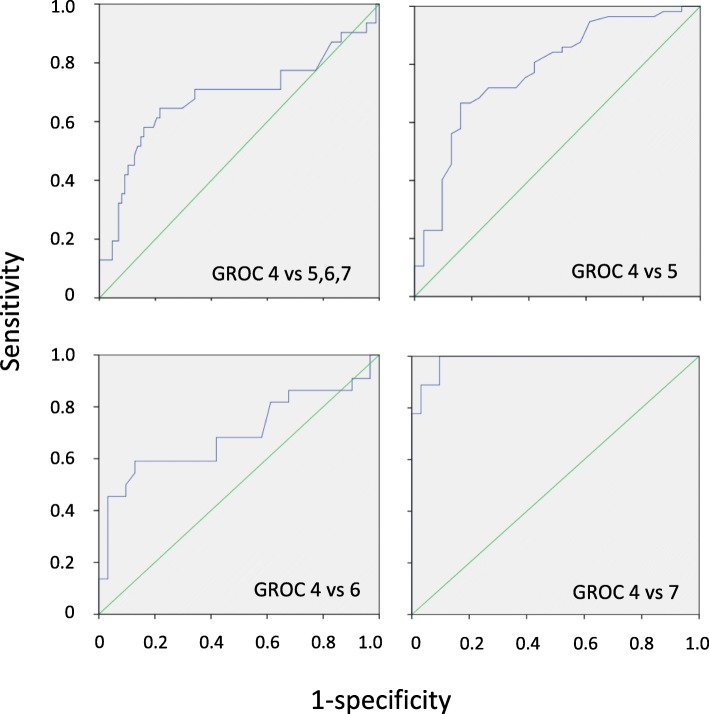
Receiver operating characteristic curves between the stable and improved groups

The ROC curves of the stable group versus the improved group and individual analysis between the stable group and small, medium and large improver groups are shown in Fig. 2. Table 4 includes the AUC, MIC with sensitivity and specificity analysis between the stable and improved groups. All AUC values were significant and within acceptable proximity to the 0.70 cut-off.

**Table 4:** Area under the curve (AUC) and minimum important change (MIC) with sensitivity and specificity

Stable group	Improved groups	AUC	95% CI		MIC	Sensitivity	Specificity
GROC 4 (*n* = 31) vs	GROC 5, 6, 7 (*n* = 88)	0.68*	0.55	0.81	12.30	0.65	0.78
	GROC 5 (*n* = 57)	0.77*	0.67	0.88	6.5	0.67	0.84
	GROC 6 (*n* = 22)	0.69*	0.54	0.85	8.5	0.59	0.87
	GROC 7 (*n* = 9)	0.98*	0.96	0.99	11.15	0.99	0.99

*Significance *p* < 0.05

## Discussion

The SPADI was successfully translated and cross-culturally adapted into Nepali using the recommended international guidelines [8]. The excellent comprehensibility and ease of completion of the Nepali translated version of the SPADI questionnaire were reinforced by the feedback from the participants included in the pre-testing phase of this study and reflect the appraisal of the original English version in this regard [4]. The minor adaptions such as changing of the measurement units to metric and words or phrases such as dress items to commonly used equivalent Nepali terms were reported by participants to contribute to the comprehensibility of the SPADI-NP.

The SPADI-NP also demonstrated excellent validity and reliability and acceptable responsiveness for Nepali participants with shoulder pain. The factor analysis in the current study suggested the items of the SPADI-NP were loaded under two factors and largely followed the structure confirmed in the original English version of pain and disability [23, 24]. However, three pain items (item 3—reaching for something on a high shelf?, item 4—touching the back of your neck?, item 5—pushing with the involved arm?) and a disability item (item 12—carrying a heavy object of 5 kg?) were loaded in both factors, and it does suggest that the differentiation between the two factors may be difficult in some circumstances. This may be due to individuals identifying pain as the limiting factor in task completion and making it not possible to separate pain and functional limitation/disability. This is the likely reason why previous studies have reported more of a unidimensional structure for the SPADI, especially where the pain construct is very high in conditions such as in adhesive capsulitis [25, 26]. A recent large randomised control trial and a multi-centre cohort study have provided further evidence of a two-factor structure for the SPADI, but even these studies suggest that the two factors of pain and disability are not well delineated with some items cross-loading [27, 28] and misfit of the disability items when the Rasch model is used [28].

The internal consistency calculations in this study were based on the two-factor structure and demonstrated good results. The subscales and total SPADI-NP results are similar to the original English version and other translated versions [3, 5, 7], indicating the items that measure the same construct (i.e. pain or disability) produce similar scores and therefore are providing a reliable measure for that construct. The SPADI-NP also maintains its reliability between administration mode (interview and self-report) with excellent ICC scores for the two constructs and the overall test. However, as there are some inconsistent results of factor analysis reported in the literature, the subscale analyses of internal consistency and test-retest reliability needs to be interpreted cautiously.

The validity of the SPADI-NP was supported by confirmation of the a priori hypotheses with high positive correlation of the disability items and a moderate positive correlation between the pain items of the SPADI-NP and DASH-NP. This further suggests that the SPADI-NP is able to measure the constructs that it is intended to measure, i.e. pain and disability. Interestingly, the strength of correlation between the disability items is higher than pain. This may be the result of the higher number of disability items in the DASH-NP, giving a more comprehensive analysis, or alternatively, it could again reflect the difficulty of distinguishing pain from functional activities. The numerical scale used in the SPADI (in contrast to the DASH worded scale) was also flagged in the pretesting as a possible problem for rural participants. However, these results seem to confirm that the translated SPADI performed well for all participants.

Responsiveness as measured by the area under the curve of the ROC was shown to be significant and within acceptable proximity to the proposed cut-off of 0.70 for all comparisons between the stable and improved groups. Although this value was lower than the original SPADI (AUC 0.87) [4], it still indicates that the SPADI-NP is able to distinguish between stable and improved participants. An MIC value of 12.3 (out of 100) for the SPADI-NP was also within the range (8–13.2) reported for the original English version [2], but most importantly, in the current study, the MIC was larger than the MDC and therefore represents a true minimal change value and supports the validity of the instrument.

Results of the current study showed no significant difference between the mean scores of participants completing the SPADI-NP by self-report or interview method and between face-to-face interviews at a hospital or over the phone. This evidence supports the a priori hypothesis that administration of the SPADI-NP by self-report, phone interview and face-to-face interview are all appropriate methods of administration without compromising its validity. In addition to the rigorous cross-cultural adaption process, the COSMIN checklist recommends studies should provide some form of analysis to demonstrate representativeness of the translated PROM in the new cultural setting. This can include different analyses between groups of individuals based on ethnicity, gender, socioeconomic background, education levels etc. In the current study, we chose to compare the efficacy of administering the questionnaire by interview or self-completion. This was to validate the versatility of the SPADI-NP in the Nepali culture where although it is improving, poor education levels and low literacy rates remain an issue. An additional problem in Nepal is the remoteness of living and difficulty of individuals to attend follow-up appointments. Therefore, for a questionnaire to be inclusive and useful to a wide range of individuals in Nepal, it is imperative that it is able to be administered by interview (in person or over the phone) or self-reporting. The SPADI-NP meets this important criterion and increases its versatility for both rural and urban Nepali populations.

The strengths of this study were the careful translation process undertaken, diverse cohort and the thorough statistical analysis adopted from the latest COSMIN recommendations for the assessment of PROMs [9]. The use of the GROC scale as an indicator of ‘overall improvement’ instead of improvement in pain and/or disability may be considered a limitation of this study and could explain the lower than expected responsiveness value in this sample. Ideally, to consider it as a criterion, both the measures should assess the same construct (i.e. pain and disability). However, using GROC to assess the overall improvement is a common practice [11, 13, 14] and is recommended by the COSMIN guidelines to dichotomise participants into stable and improved groups [9, 22].

## Conclusion

The Nepali version of the SPADI demonstrated excellent comprehensibility, reliability, validity and acceptable responsiveness. It also proved its versatility in the method of administration by both self-report and interview (phone and face-to-face) in a wide range of Nepali shoulder pain participants. Therefore, the SPADI-NP would be recommended to assess individuals in Nepal with shoulder pain and disability in both clinical practice and research settings.

## Data Availability

The datasets used and/or analysed during the current study are available from the corresponding author on reasonable request.

## Abbreviations

AAOS: American Association of Orthopaedic Surgeon
AUC: Area under the curve
COSMIN: COnsensus-based Standards for the selection of health Measurement INstruments
DASH: Disability of Arm, Shoulder and Hand
GROC: Global Rating of Change
ICC: Interclass coefficient correlation
KMO: Kaiser-Meyer-Okin
MDC: Minimal detectable change
MIC: Minimal importance change
PROM: Patient-reported outcome measure
ROC: Receivers operating curve
SD: Standard deviation
SEM: Standard error of measure
SPADI: Shoulder Pain and Disability Index
VAS: Visual analogue scale
